# On Differential Imaging Using Electromagnetic Simulation for Vehicular Antenna Signature Analysis

**DOI:** 10.3390/s21113796

**Published:** 2021-05-30

**Authors:** Jose Antonio Solano-Perez, María-Teresa Martínez-Inglés, Jose-Maria Molina-Garcia-Pardo, Jordi Romeu, Lluis Jofre-Roca, Christian Ballesteros-Sánchez, José-Víctor Rodríguez, Antonio Mateo-Aroca, Raúl Guzmán-Quirós

**Affiliations:** 1 Departamento Tecnologías de la Información y las Comunicaciones, Universidad Politécnica de Cartagena, Cartagena, 30202 Murcia, Spain; josemaria.molina@upct.es (J.-M.M.-G.-P.); jvictor.rodriguez@upct.es (J.-V.R.); raul.guzman.quiros@gmail.com (R.G.-Q.); 2 Centro Universitario de la Defensa, Universidad Politécnica de Cartagena, Base Aérea de San Javier, Academia General del Aire, 30720 Murcia, Spain; mteresa.martinez@cud.upct.es; 3 CommSenslab, Department of Signal Theory and Communications, School of Telecommunications Engineering, Technical University of Catalonia (Universitat Politecnica de Catalunya, UPC) Campus Nord UPC, Edif, D-3 Jordi Girona, 1-3, 08034 Barcelona, Spain; romeu@tsc.upc.edu (J.R.); jofre@tsc.upc.edu (L.J.-R.); christian.ballesteros@tsc.upc.edu (C.B.-S.); 4 Departamento Automática, Ingeniería Eléctrica y Tecnología Electrónica, Universidad Politécnica de Cartagena, Cartagena, 30202 Murcia, Spain; Antonio.Mateo@upct.es

**Keywords:** electromagnetic simulation, terahertz, differential imaging, differential currents

## Abstract

The current trend in vehicles is to integrate a wide number of antennae and sensors operating at a variety of frequencies for sensing and communications. The integration of these antennae and sensors in the vehicle platform is complex because of the way in which the antenna radiation patterns interact with the vehicle structure and other antennae/sensors. Consequently, there is a need to study the radiation pattern of each antenna or, alternatively, the currents induced on the surface of the vehicle to optimize the integration of multiple antennae. The novel concept of differential imaging represents one method by which it is possible to obtain the surface current distribution without introducing any perturbing probe. The aim of this study was to develop and confirm the assumptions that underpin differential imaging by means of full-wave electromagnetic simulation, thereby providing additional verification of the concept. The simulation environment and parameters were selected to replicate the conditions in which real measurements were taken in previous studies. The simulations were performed using Ansys HFSS simulation software. The results confirm that the approximations are valid, and the differential currents are representative of the induced surface currents generated by a monopole positioned on the top of a vehicle.

## 1. Introduction

Current vehicle design relies on the placement of different sensors and antennae operating in frequency bands from 3 GHz up to millimetric waves (mmWave) for different purposes such as communications, sensing, and/or positioning. It is anticipated that the number of antennae and sensors installed on the vehicle surface will increase in future generations of vehicles as 5G-generation capabilities come to the fore [1]. The integration of sensors and antennae within the same vehicular platform is a complex task because the radiation pattern of these elements is influenced by the platform structure, other sensors and antenna installed, and how the induced surface current is distributed across the structure of the vehicle. Consequently, there is a requirement to assess the effects that the vehicular structure has on the radiation properties of the antennas and implement co-location strategies that minimize mutual interaction between them. There are two methods to achieve these objectives: Via a full-wave electromagnetic simulation or via experimental methods. In this context, experimental methods rely on complex measurement tests in which non-invasive techniques that minimize the impact on the results during the measurement process with the test probes are desired. Electromagnetic simulation methods combine modeling and characterization with high-performance computational and time-consuming processing; however, the resulting error depends on the simulation algorithm and the accuracy of the electromagnetic model and its characterization.

Differential imaging represents an alternative to experimental and simulation methods. This novel concept was presented in [2] as a measuring technique that is based on the use of proven imaging systems [3] and prior measurements. The differential imaging and differential currents concepts represent a novel approach by which it is possible to estimate the antenna signature without influencing the measurements, in other words, without altering the surface current distribution or electric field distribution generated by the antenna. This study aimed to explore the approximations and assumptions that underpin differential imaging and currents by performing full-wave electromagnetic simulations to verify this novel concept.

Ansys HFSS has been employed for full-wave simulations of antenna and current distribution in a variety of contexts and electromagnetic problems such as the one here studied [4]; Ansys HFSS currently represents one of the most reliable tools in the market of general purpose full-wave electromagnetic simulation. For example, in [5], Ansys HFSS software was used to optimize a sensor, and the results indicated that the software represented a reliable means of electromagnetic simulation. In another example, in [6], the software was once again found to perform effectively in simulating the performance of a dual-band antenna.

Between some of the main contributions that define the most recent state-of-the-art on differential imaging is [7], where the differential imaging concept is used for observing a scenario along a time interval for analyzing the images. Additionally, there is [8], where the differential imaging approach is applied for providing 3D images using a microwave system. Finally, in [9], the review presents time differential images as a method to exploit imaging capture by ultra-wideband microwave imaging sensors.

The aim of the research outlined in this paper was to perform full-wave electromagnetic simulations to verify the differential imaging concept when estimating the surface current distribution generated by a monopole antenna placed on a vehicle surface and near electric fields evaluated in a plane. The electromagnetic simulation was performed on a metallic scale car with an electrical length of 100 wavelengths at 300 GHz, which was externally illuminated by a plane wave from the top to replicate the conditions tested in [2]. This simulation was designed to confirm the approximations and assumptions performed in the development of the differential currents concept. Additionally, the analysis of the differential in near electric field component simulations verified the surface current distribution across the car structure metallic surface that was generated by the monopole.

The remainder of this paper is organized as follows. Section 2 explains the rationale associated with differential imaging before progressing to outline the approximations and assumptions that were applied in the current study to obtain the current distribution associated with the antenna signature by means of full-wave electromagnetic simulation. This section also outlines the simulation details and parameters. Section 3 presents the simulation results in terms of the surface current distribution, near electric field representation, and differential currents and electric fields. Section 4 discusses the results related to the different approximations and differential concept. Finally, Section 5 presents the conclusions that can be drawn from the study and summarizes the contribution that the research makes to the existing understanding of differential imaging.

## 2. The Differential Imaging Technique

This section presents an overview of the differential imaging process employed to reconstruct and assess the currents generated by a radiating antenna placed on top of a vehicle. It explains the use of electromagnetic simulations to identify the key assumptions that underpin differential imaging [2].

The decomposition of antenna scattering during structural scattering and the scattering of the radiation mode of an antenna has been the subject of a previous study [10]. The goal of the current study was to reconstruct the currents associated with structural scattering to develop the concept of differential imaging and differential currents with the underlying intention of identifying methods to optimize the co-location of various antenna systems across a vehicle surface.

The innovative differential imaging technique presented in [2] involves the use of external illuminating geometry to obtain the currents created by a vehicle monopole antenna on the vehicle surface, without invasive proof to obtain measurements. The implementation of the external illumination geometry is performed by means of a Vector Network Analyzer (VNA) connected to a horn antenna that works as a transmitter (Tx) and another horn antenna that works as a receiver (Rx) to measure the scattering parameters at specific frequencies [2,3]. The polarization of both Tx and Rx horn antennas is linear.

The currents created by the monopole antenna placed on the vehicle can be understood as the differential contribution derived from two different states: vehicle antenna ON and OFF states. The ON state arises when the antenna is in a closed circuit, i.e., the feed point is connected to the metallic structure of the vehicle, while the OFF state arises when there is no functioning antenna or when the antenna is in an open circuit. The differential contributions of vehicle antenna radiation are obtained by evaluating the scattering fields generated by the currents on the surface in the two different states: the ON state and the OFF state [11].

When the vehicle antenna is in the ON state, the total scattered fields ${\vec{E}}_{antenna-ON}^{total}$ are produced by scattering fields composed of the three terms, as follows:

- Currents generated on the vehicle surface by external illuminating geometry ${\vec{E}}_{antenna-ON}^{external-illumination}$,
- Structural currents generated into the vehicle antenna structure without interaction with antenna port: ${\vec{E}}_{antenna-ON}^{antenna-structural}$,
- Radiating mode of the antenna, ${\vec{E}}_{antenna-ON}^{antenna-radiating}$.

Then, the ${\vec{E}}_{antenna-ON}^{total}$ is as follows [12]:

(1) $${\vec{E}}_{antenna-ON}^{total}={\vec{E}}_{antenna-ON}^{external-illumination}+{\vec{E}}_{antenna-ON}^{antenna-structural}+{\vec{E}}_{antenna-ON}^{antenna-radiating}$$

The next state is when the vehicle antenna is in the OFF state. The total scattered fields ${\vec{E}}_{antenna-OFF}^{total}$ are generated by the contributions of the scattered fields due to:

- Currents created on the vehicle surface by the external illuminating geometry ${\vec{E}}_{antenna-OFF}^{external-illumination}$
- Structural currents created into the vehicle antenna structure without interaction with its port ${\vec{E}}_{antenna-OFF}^{antenna-structural}$.

Then, the ${\vec{E}}_{antenna-OFF}^{total}$ is as follows:

(2) $${\vec{E}}_{antenna-OFF}^{total}={\vec{E}}_{antenna-OFF}^{external-illumination}+{\vec{E}}_{antenna-OFF}^{antenna-structural}$$

Compact vehicle antennas are installed in the majority of vehicles that use resonant antennas. As such, the following approximations should be taken into consideration:

- Higher-order interactions, for instance, multiple reflections between the external illuminating geometry and the vehicle antenna or the vehicular platform, may be neglected since they are much smaller than the other interactions.
- The scattered field produced by the unchanged parts, such as the vehicular platform and the structure of the antenna; for both states of the vehicle antenna is approximately the same: ${\vec{E}}_{antenna-ON}^{external-illumination}≅{\vec{E}}_{antenna-OFF}^{external-illumination}$ and ${\vec{E}}_{antenna-ON}^{antenna-structural}≅{\vec{E}}_{antenna-OFF}^{antenna-structural}$.

Considering the previous approximation, it could be established that the reconstructed currents above the vehicle surface obtained from the differential scattered fields ${\vec{E}}_{antenna}^{differential}$;

(3) $${\vec{E}}_{antenna}^{differential}={\vec{E}}_{antenna-ON}^{total}-{\vec{E}}_{antenna-OFF}^{total}≅{\vec{E}}_{antenna-ON}^{antenna-radiating}$$

The theoretical basis of the technique presented above relies on two approximations: “multiple reflections between the external illuminating geometry and the vehicle monopole antenna or the vehicular platform, may be neglected” and “the scattered field produced by the unchanged parts like the vehicular platform and the structure of the monopole antenna, for both states of the vehicle antenna are approximately the same.” Both statements are reasonable. To assess the effect of such approximations, electromagnetic simulations in combination with insights into the mutual impedance elements could make it possible to demonstrate the concept by quantifying the effects.

In terms of the first assumption, “multiple reflections between the external illuminating geometry (Tx antenna) and the vehicle monopole antenna or the vehicular platform may be neglected”, the measurement setup uses directive antennas that contribute to the removal of reflections. The use of a monopole antenna in the vehicle oriented in the direction of the Tx antenna is useful because even when the excitation of the monopole mode may be reduced, its mutual coupling with the Tx antenna is also reduced. As such, the multiple reflections may be neglected while the surface currents on top of the vehicle due to the antenna are simultaneously visible, especially when the Tx moves away from the perpendicular direction. The contributions of multiple reflections are already very attenuated, as they lose almost all their energy; as such, the impact they have on the results should be assumed to be negligible. In terms of the second assumption, “the scattered field produced by the unchanged parts, like the vehicular platform and the structure of the antenna, for both states of the vehicle antenna is approximately the same”, it entails ensuring that these parts remained unaltered “on purpose” in both cases to ensure that the results show the variation due to the presence or otherwise of the antenna. This study sought to verify both assumptions that are part of the theoretical proof of the differential imaging approach through the use of simulation techniques. It was anticipated that an electromagnetic simulation based on both approximations would provide evidence to confirm these assumptions and validate the theoretical approach that underpins differential imaging.

Another specific concern associated with the differential imaging setup defined in [2], which was based on [3], is that Tx is positioned pointing from the top view of the car to the vehicle vertical monopole antenna such that the end of the antenna appears perpendicular to the polarization of the Tx antenna. This configuration may not be optimal for exciting the resonant modes of the monopole antenna on top of the car. Consequently, this configuration requires some discussion. The measurement configuration has been selected to improve the detection of the surface currents. The difference in the polarization of the Tx and Rx horns versus the polarization of the vehicle monopole antenna is clear. In any case, when the car is illuminated by the Tx with the monopole antenna in contact with the vehicular surface, the current distribution is perturbed by this antenna. It is difficult to implement an alternative approach with measurements that detect the surface current on the vehicle while simultaneously exciting the vehicle monopole antenna. The monopole antenna is oriented perpendicular to the surface illuminated by Tx/Rx antennas. Considering that monopole antenna excitation mode would be reduced, the mutual coupling between the Tx and Rx antenna with monopole is reduced too. Based on the previous consideration, multiple reflections could be neglected. However, the surface currents that the monopole antenna generates on the vehicle surface should be visible when Tx is illuminating from a perpendicular direction. It is important that these factors are taken into consideration when developing an electromagnetic simulation to verify the differential imaging concept.

### Simulation Description and Configuration

The electromagnetic simulations were performed using the HFSS (Version 2021R1) [4] commercial electromagnetic software provided by Ansys. The setup was based on that developed in [2], where a bi-static radar was implemented using a Vector Network Analyzer (VNA) connected to one head to transmit (Tx), generating the external illumination, and one to receive (Rx), in order to measure the scattering parameter, or S_21_, of a metallic car of 10 cm length (100 *λ_0_* @ 300GHz). Using the frequency-dimension scale translation, the 10 cm long metallic car under measurement was equivalent to a real vehicle of 4.3 m in length, corresponding with a frequency range between 2.5 and 3.8 GHz.

**Figure 1:**
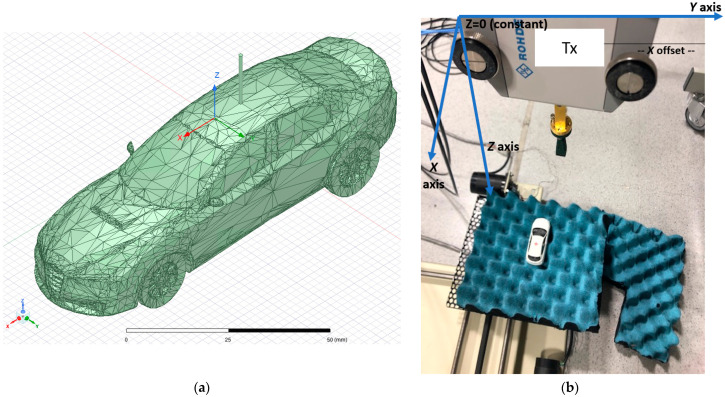
Geometry of the problem: (**a**) electromagnetic simulation arrangement including the 3D model facets; (**b**) measurement geometry arrangement used in [2].

The geometry of the car for the simulation is depicted in Figure 1. Figure 1a depicts the 3D car model used for the simulation. Figure 1b presents the measurement geometry used in [2]. The vehicle was simulated both with and without a monopole antenna of 15 mm in length placed on top of the car.

To replicate these conditions, a frequency of 300 GHz with a metallic car with an electric size of 100 wavelengths, or 10 cm in length approximately, were selected for the purpose of this simulation. The electromagnetic simulation scenario emulates the Tx horn antenna of the experiments through a plane wave excitation that impinges on perpendicular to the car topside (z-direction). Evaluation of the near electric field in a specific XY plane and the surface current distribution on the vehicle surface is then obtained for each one of the monopole scenarios (with (ON state) and without (OFF state) monopole antenna).

The object was placed on an XY plane located 0.5 m from a *Tx* horn antenna that emitted a planar wave in far-field conditions at terahertz frequencies (300 GHz) to illuminate the metallic vehicle as in [2]. The *Tx* horn antenna was considered in the far-field condition because the excitation was placed 50 cm from the vehicle (500 wavelengths at 300 GHz). The polarization of the planar wave was equivalent to that used for the pyramidal horn in [2] (i.e., along the *y* axis for the coordinate system employed for measurement and simulation setup).

The electromagnetic simulations involved a full-wave simulation of the surface currents and near electric field evaluated in a plane above the car with and without an antenna mounted on top of the vehicle roof to verify the effects of both states under excitation at 300 GHz. The Method of Moments (MoM) technique was used to solve the integral form of Maxwell’s equations. MoM is a full-wave numerical technique for solving open-boundary electromagnetic problems. For the specific electromagnetic problem defined in this paper, the boundary conditions on the surface of the vehicle were influenced by the fact that it was metallic. The results provided by the MoM facilitated an estimation of the surface current distribution and electric field in a defined plane. The MoM approach was selected because it represents a very efficient technique in terms of processing resources and time, compared with the time and frequency domain Finite Element Method (FEM) evaluated in a box containing the vehicle. Adaptive meshing was used to solve the simulation problem with 1,092,296 elements and an element length of between 0.0136 and 0.37 wavelengths.

A high-performance computer (HPC) with the following characteristics was used for these simulations:

- Intel Xeon Gold 6146 3.2 GHz—12 cores. The processing was performed using 4 cores.
- 512 GB RAM memory. The total memory used for adaptive meshing was 43.5 GB.

Although MoM reduces the processing time, it does place a significant demand on processing resources. The processing time for a 10 cm vehicle simulating at 300 GHz was 7 h using the HPC stated above for each simulation and each frequency.

## 3. Results

**Figure 2:**
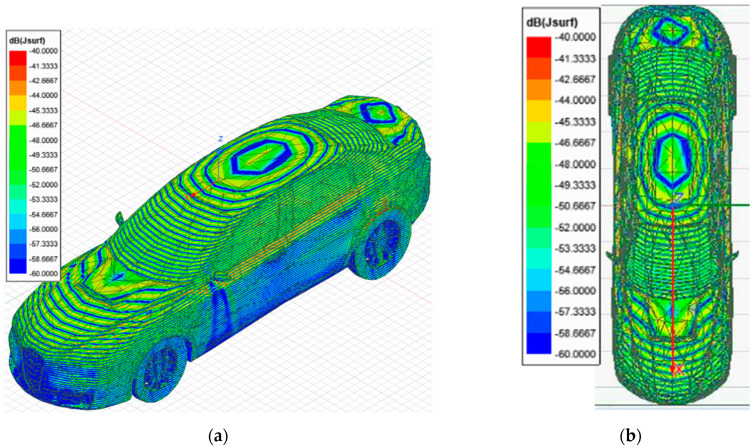
Surface current distribution imaging generated by Ansys HFSS software for a 10 cm long vehicle without antenna (OFF state), illuminated at 300 GHz: (**a**) 3D view of the surface current distribution across the vehicle; (**b**) top view of the surface current distribution.

This section presents an overview of the results of the full-wave simulation together with an interpretation of the surface current distribution and near electric field calculation in the plane above the car, at the terahertz band. The surface current distribution is displayed as the modulus of the current at a given instant, i.e., for a given phase, presenting the maximum value. The first simulation was performed to identify the surface currents at 300 GHz on a vehicle without an antenna installed on the top. This simulation replicated the OFF state. Figure 2 shows a current distribution that takes the form of a characteristic shape that is useful for imaging purposes. The surface current distribution was uniform, demonstrating that the external illuminations work properly.

**Figure 3:**
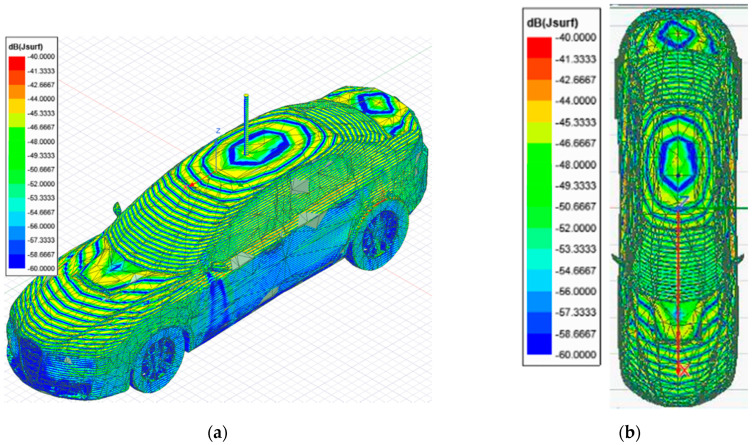
Surface current distribution imaging generated by Ansys HFSS software for a 10 cm long vehicle with antenna (ON state), illuminated at 300 GHz: (**a**) 3D view of the surface current distribution of the vehicle; (**b**) top view of the surface current distribution.

The surface currents at 300 GHz across a vehicle with a monopole antenna installed at the top were simulated (ON state). Figure 3 presents the current distribution across the car surface and in the monopole after the car is illuminated.

**Figure 4:**
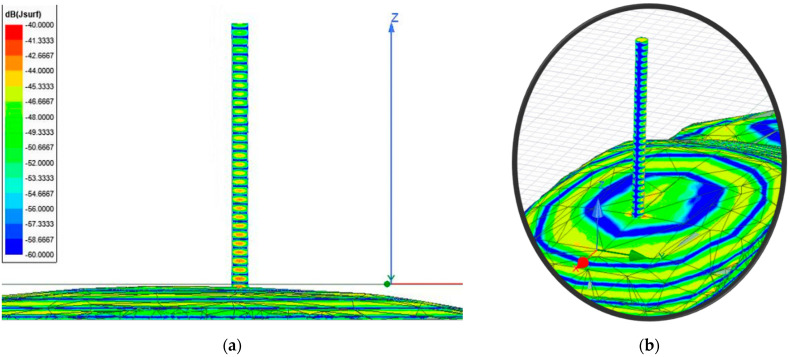
Surface current imagery distribution generated by Ansys HFSS software in the monopole installed on a 10 cm long vehicle illuminated at 300 GHz: (**a**) lateral view of the monopole antenna showing the surface current distribution; (**b**) magnification of the monopole antenna to show the current distribution across the surface.

Figure 4 shows the surface currents induced along the short-circuited 15 mm monopole stacked on top of the car. As expected, the surface currents induced on the surface of the car because of the impinging 300 GHz plane wave excite the monopole from its bottom, presenting a surface current distribution form along which present 30 peaks, i.e., 15 wavelengths, typical of the monopole resonance with such electrical length. As such, the antenna should produce a radiation equivalent to a monopole of 15 *λ_0_* length at 300 GHz with minimum (null) at zenith and secondary lobes looking off-broadside.

**Figure 5:**
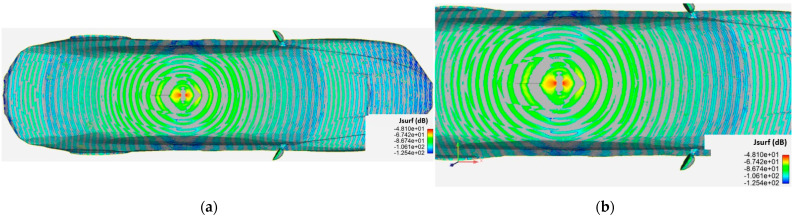
Differential surface current distribution imagery between the two simulation cases on a logarithmic scale generated by Ansys HFSS software and displayed using Ansys ENSIGHT: (**a**) distribution across the entire vehicle; (**b**) magnified view of the distribution at the top of the vehicle where the monopole was installed.

The difference between the simulated surface current across the two (ON / OFF state) cases is presented in Figure 5. The differential surface current distribution shows the antenna signature in a logarithmic scale to improve the current distribution differences. As can be observed, maximum delta between the current distribution pattern is observed around the currents induced around the position of the monopole location on top.

**Figure 6:**
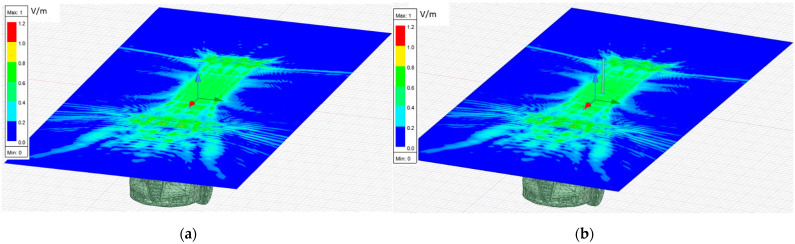
Pictorial representation of the near electric field on a plane in contact with the top of the car for the two simulation cases generated by Ansys HFSS software: (**a**) vehicle without an antenna installed at the top (OFF state); (**b**) vehicle with a monopole installed on the top (ON state).

The near electric field evaluated on a plane parallel to the car in contact on top of the vehicle when the vehicle was illuminated at 300 GHz, both with and without antenna, was simulated to observe the difference in the electric field generated between both cases. Figure 6 presents the position of the plane related to the car depicting the near electric field distribution in module, considering the three components of the electric field.

**Figure 7:**
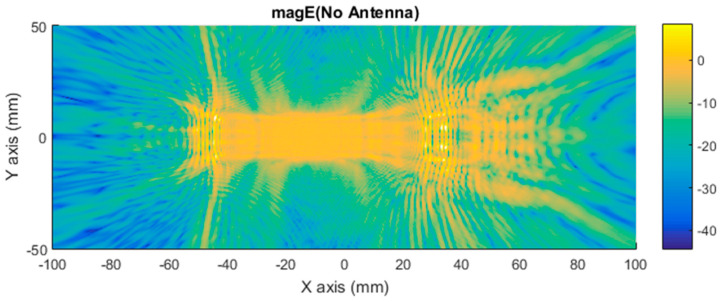
Distribution of near electric field magnitude (in logarithmic scale) of the three components without antenna (OFF state), evaluated in a plane in contact with the top of the car, illuminated at 300 GHz. Figure generated by MATLAB software using the complex near electric field raw data obtained at each point of the plane based on a simulation using Ansys HFSS software.

The simulations of the near electric fields provide three vectorial components of the electric fields in complex format. The total electric field vector is the sum of the three vectorial components for each point of the plane. After that, the modulus of the total electric field that it is displayed in the different figures is calculated. For the following figures, the electric field is represented in terms of the magnitude (module) of the three components and in the logarithmic scale to improve the representation. Figure 7 shows the total electric field vector (three components) in magnitude (vector module) evaluated in a plane for the case without an antenna. It shows how the near electric field is distributed when the car is illuminated. Scattered fields are presented in this figure as the main items for imaging and differential imaging.

**Figure 8:**
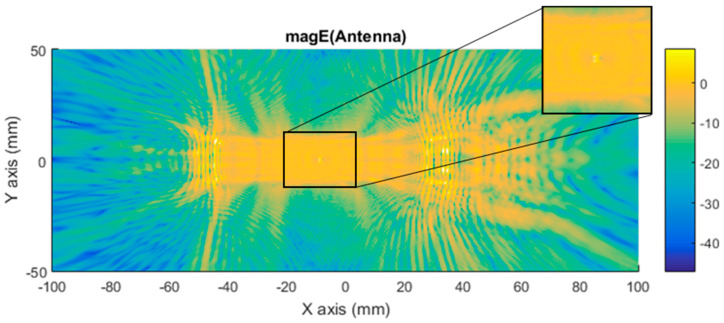
Distribution of near electric field magnitude (in logarithmic scale) of the three components with antenna (ON state), evaluated on a plane in contact with the top of the car, illuminated at 300 GHz. The region where antenna monopole diffraction pattern is identified is zoomed. Figure generated by MATLAB software using the complex near electric field raw data obtained at each point of the plane based on a simulation using Ansys HFSS software.

Figure 8 shows how the near electric field was distributed when a car with a monopole installed on top was illuminated. The antenna position is identifiable if the Figure 8 is compared with Figure 7. Then, it can be observed just at the middle of Figure 8, close to (0,0), where there is a glowing point on top of the car roof where a peak on the antennae-field diffraction pattern can be identified because of the surface currents around the monopole contribution.

**Figure 9:**
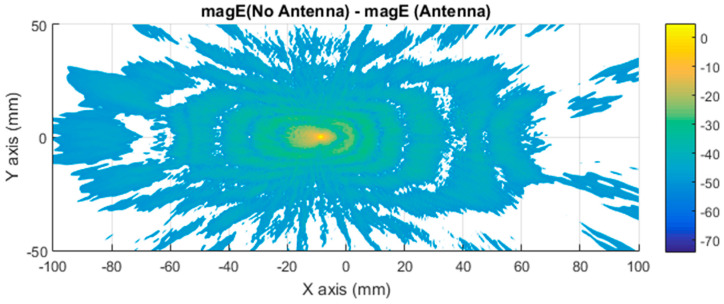
Distribution of near electric field difference between a vehicle with and without antenna magnified (in logarithmic scale) for the three components evaluated across a plane in contact with the top of the car, illuminated at 300 GHz.

The differential contribution, evaluated in terms of electric field vector magnitude (module of the three components of an electric field, in logarithmic scale for improving the representation) between the car with and without antenna, is presented in Figure 9. This figure confirms the differential contribution to identify the antenna signature, or in other words, the current distribution associated with the near electric field generated by the antenna in the vehicle surface. The representation of the near electric field replicated the electric field generated by a monopole in a plane.

**Figure 10:**
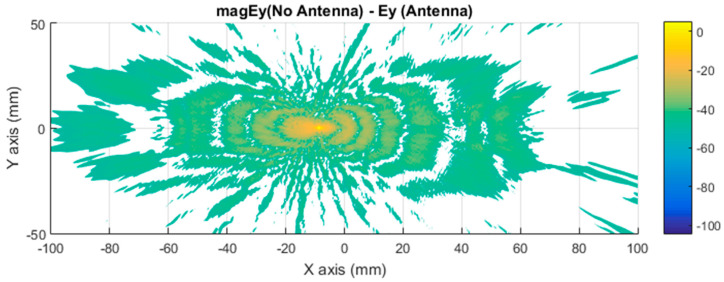
Distribution of near electric field difference between a vehicle with and without antenna in magnitude (in logarithmic scale) for the Y axis component, evaluated on a plane in contact with the top of the car, illuminated at 300 GHz.

**Figure 11:**
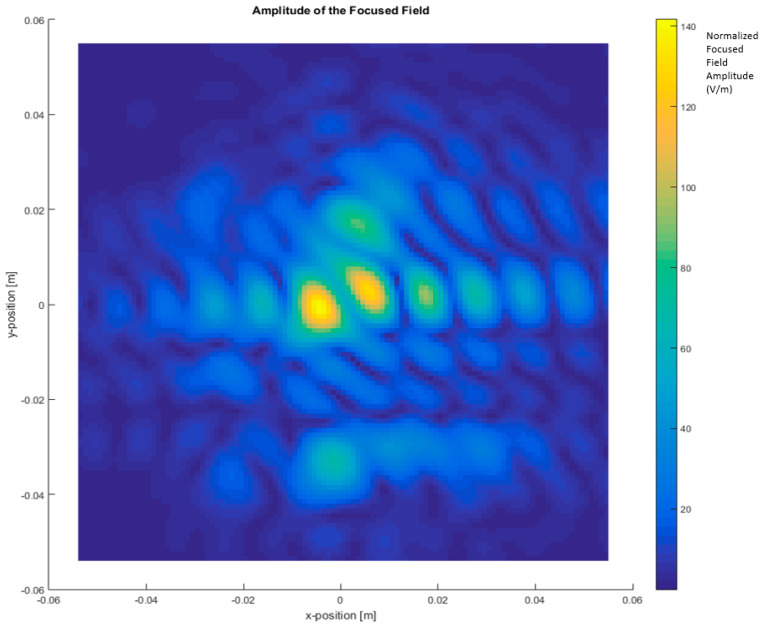
Differential focused field amplitude between the metallic car with and without an antenna. Frequency range: 295 GHz to 305 GHz (BW = 10 GHz) [2].

Further simulations were performed to facilitate a qualitative comparison between the differential imaging obtained by the simulation and the results obtained in [2]. The measurements were taken using a VNA connected to a Tx pyramidal horn antenna and Rx pyramidal horn antenna. The difference in the near electric field in the Y axis between the car with antenna and without antenna, presented in Figure 10, was compared with the previous experimental results, presented in Figure 11. The Y component of the electric field was selected because the maximum response of the horns and polarization occurs in that axis.

Figure 10 presents a representation of the module electric field at 300 GHz in logarithmic scale for the component in the Y axis. Figure 11 is the normalized electric field calculated by the imaging multifrequency bi-focusing algorithm [3] using the scattering fields measured by the VNA through a bandwidth of 10 GHz around a central frequency of 300 GHz. After stating the nature of the condition of each figure in both figures, the maximum radiation generated by the monopole and a secondary maximum on one side were identified. 

**Figure 12:**
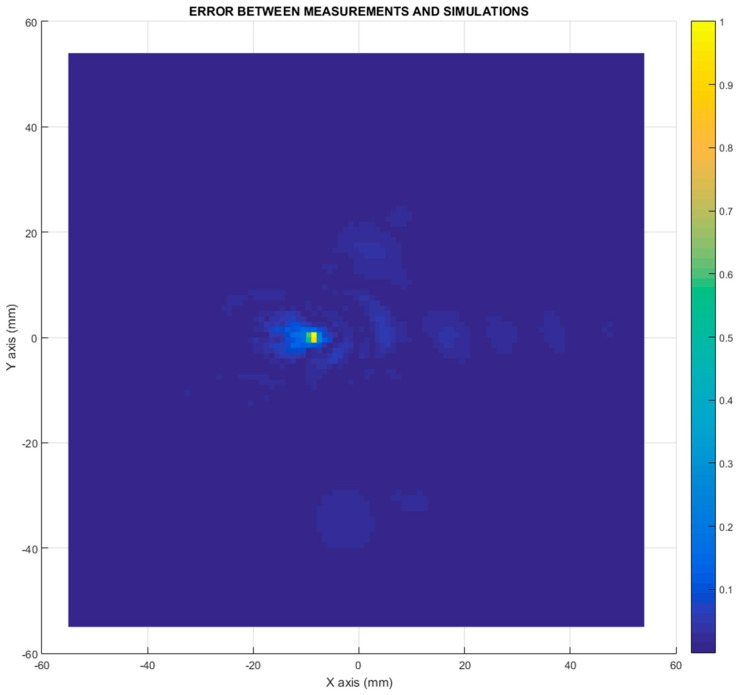
Error between the differential imaging measurement and the differential imaging simulations.

The error between the differential imaging measurements and the simulations is defined in terms of the module of the difference in electric field. The differential imaging measurements and the simulations have to be normalized to the maximum value before calculating the difference. Then, the error is expressed in adimensional units. Figure 12 compares the differential imaging measurements with the simulation measurements. The simulation error data were amplified, calibrated, and normalized to the maximum to enable this comparison. The differential imaging measurements were normalized to the maximum too. The error distribution shown in Figure 12 depicts the error in the position of the monopole antenna. This error can be attributed to the misalignment between the 3D model and the real measurement in combination with the effects associated with the multifrequency imaging algorithm.

**Figure 13:**
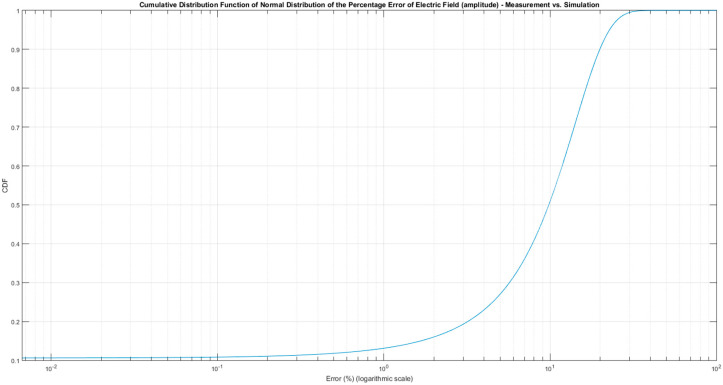
Cumulative Distribution Function (CDF) of Normal Distribution of the Percentage Error of Electric Field (amplitude) in logarithmic scale in X axis.

In order to provide a complete comparison of measurement [2] and simulation results, the error evaluation in terms of the Cumulative Distribution Function (CDF) of Normal Distribution of the Error of Electric Field calculated as the percentage error of the electric field amplitude was included. Figure 13 shows the distribution of the percentage error, depicting that the 90% of the points presents an error in amplitude less than 19.9%, after adjusting the levels of the simulations to the measurements to be comparable.

Another important parameter to evaluate the error is the Root Mean Square Error (RMSE) of the percentage error of the electric field amplitude. The RMSE value is 12%.

## 4. Discussion

This section examines the extent to which the assumptions and approximations applied within differential imaging and differential current methods to estimate the currents induced by a monopole (or any antenna) in the car surface are valid. The aim of the full-wave electromagnetic simulation described in this paper was to verify the differential current approach for estimating the current distribution of an antenna with the underlying intention of demonstrating the reliability of the differential imaging concept. The differential current distribution presented in Figure 5 demonstrates that a differential current was obtained from the difference between the two simulation cases. This difference is representative of the antenna signature.

The first approach involves neglecting the multiple reflections among the illumination and vehicle structure. Figure 7 and Figure 8 depict the near electric fields representation for both states (OFF and ON states), confirming that the reflection in the borders caused some diffraction, but the position of Tx and Rx served to minimize and attenuate the reflections received when the scattered fields were measured. The shape of the vehicle could be identified. In addition, another concern relates to the position of the external illumination in relation to the monopole. This configuration could raise some doubts about whether it is possible to excite the monopole. Figure 3 and Figure 4 confirm that the monopole presented induced currents that generated electric fields. As such, the monopole was operating using external illumination. Moreover, the current distribution shown in Figure 2 and Figure 3 confirm that the external illumination placed at 0.5 m at 300 GHz was enough to induce surface currents across all the exposed surfaces.

The second assumption was that the scattered fields produced for both states (with and without antenna) would be approximately the same. Figure 7 and Figure 8 present the magnitude of the near electric field vectors (three components) for both states. As can be observed, the shape was almost the same in both cases. Additionally, the difference in the near electric field is characteristic of the shape of the electric fields generated by a monopole. The main difference is that the antenna could be seen in Figure 8. After the assumptions were evaluated, a qualitative comparison between the differential imaging obtained in [2] and the simulations presented in the current study was performed. Although the nature of both measurements was not the same, the shape of the differential imaging and differential currents and fields were close to each other, thereby identifying the maximum area of the induced currents and fields.

Related to the error presented in Figure 12, the level of error was evaluated. This error could be attributed to differences and misalignment between the measurements and the simulations, combined with the use of an imaging algorithm that sums the contribution of the wide frequency band.

To evaluate the error, the percentage error in terms of CDF and RMSE was evaluated. The CDF, shown in Figure 13, states that error distribution and a value around 18%, giving enough confidence to provide a distribution of the current distribution in a surface car. The current distribution shape would not be affected or deformed by an error of 18% in amplitude. Related to the RMSE, the value of 12% confirms that the error level is controlled, and it allows us to use the measurement results for estimating the surface current distribution on a car surface.

## 5. Conclusions

The full-wave electromagnetic simulations performed in the current study demonstrate the reliability of the novel differential imaging concept presented in [2]. Differential currents provide a tool for estimating the antenna signature by means of measuring the vehicle with and without antenna and calculating the differential imaging or differential currents by measuring the scattered fields. The full-wave electromagnetic simulations of the surface currents and near electric fields generated by a 10 cm miniature length metallic vehicle structure impinged by a normal plane wave can generate insights that contribute to a confirmation of the differential imaging concept and the assumed approximations. The Method of Moment approach offered by Ansys HFSS commercial software [4] was used effectively to model and solve this problem, optimizing processing time of the simulations. The comparison of the measurement [2] and simulation results were performed and evaluated in terms of CDF and RMSE to have an estimation of the error, confirming that the results are good enough for exploitation in terms of providing a shape of the surface current distribution in the car.

The findings outlined in this paper will provide a meaningful contribution to existing understanding and insight of the differential imaging method that allows researchers to obtain and observe the current distribution induced on the object without measurement probes that influence and induce error on the field measurement. Thus, this work provides additional verification of the differential imaging concept through a full-wave electromagnetic simulation.

After the full-wave electromagnetic simulation, the differential currents approach as a means of estimating the currents induced by the monopole is a step forward. Subsequently, the differential concept could be used to review and optimize the co-siting of different sensors and antennae on a vehicle.

## Data Availability

The data presented in this study are available on request from the corresponding author. The data are not publicly available due to the raw data size and format of the electromagnetic simulation.
